# Supplementary material for: In Silico and In Vitro Search for Dual Inhibitors of the Trypanosoma brucei and Leishmania major Pteridine Reductase 1 and Dihydrofolate Reductase
Source: Molecules. 2023 Nov 10;28(22):7526. doi: 10.3390/molecules28227526 (PMC10673058; doi:10.3390/molecules28227526)
Supplement: Supplementary file 1 [file molecules-28-07526-s001.zip › molecules-2660954-supplementary.pdf]

Supplementary Materials

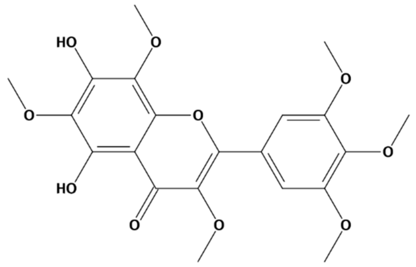

23
Phytolab
4CMJ (-8.76 kcal/mol)
5JDI (-9.62 kcal/mol)

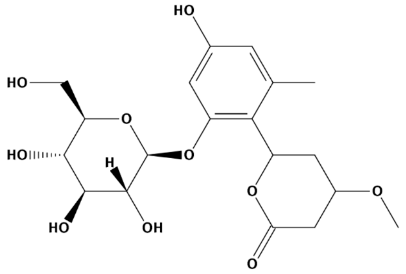

24
Phytolab
4CMJ (-9.04 kcal/mol)

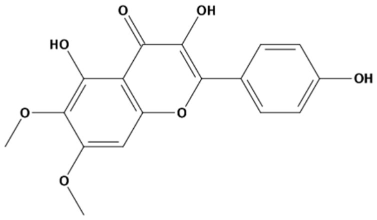

25
Phytolab
3MCV (-8.21 kcal/mol)

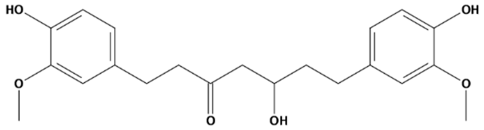

26
Phytolab
5JDI (-10.21 kcal/mol)

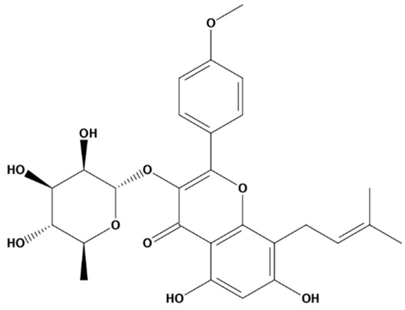

27
Phytolab
4CMJ (-8.89 kcal/mol)

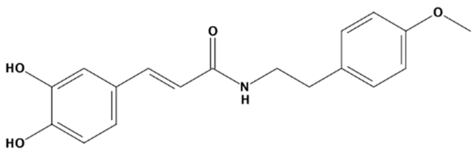

28
Phytolab
1W0C (-7.03 kcal/mol)

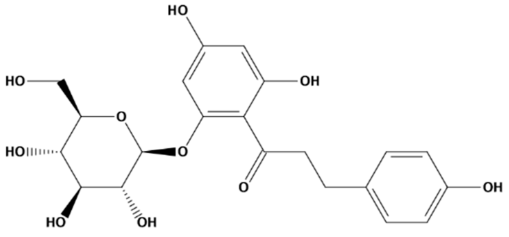

29
Phytolab
2X9G (-8.86 kcal/mol)
4CMJ (-9.11 kcal/mol)
4CMK (-8.83 kcal/mol)
3QFX (-8.29 kcal/mol)

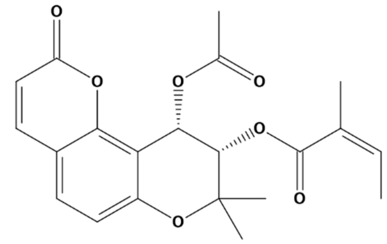

30
Phytolab
4CMK (-8.31 kcal/mol)

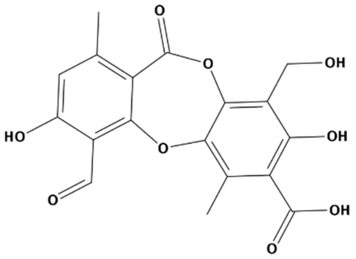

31
structural analog to top hit from
AnalytiCon Discovery
1E7W (-8.64 kcal/mol)
2BFM (-8.32 kcal/mol)
2QHX (-8.82 kcal/mol)

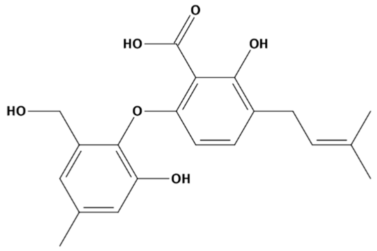

32
AnalytiCon Discovery
5JDI (-8.85 kcal/mol)

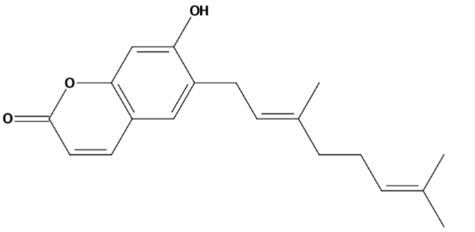

33
AnalytiCon Discovery
3MCV (-8.26 kcal/mol)

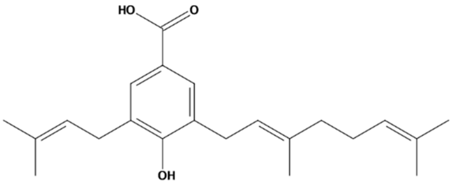

34
AnalytiCon Discovery
3MCV (-8.51 kcal/mol)
3QFX (-8.00 kcal/mol)

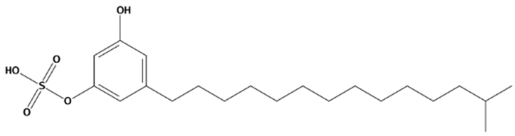

35
AnalytiCon Discovery
3QFX (-8.71 kcal/mol)

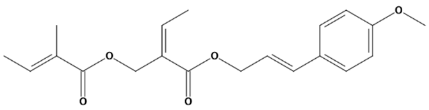

36
AnalytiCon Discovery
4CMK (-8.51 kcal/mol)

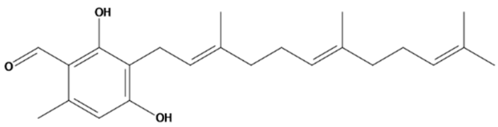

37
AnalytiCon Discovery
4CMJ (-8.23 kcal/mol)

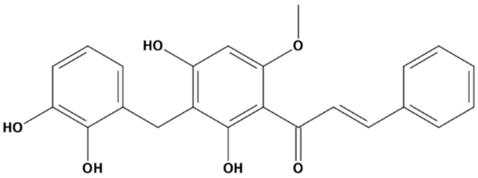

38
AnalytiCon Discovery
4CMJ (-8.47 kcal/mol)
5JDI (-9.40 kcal/mol)

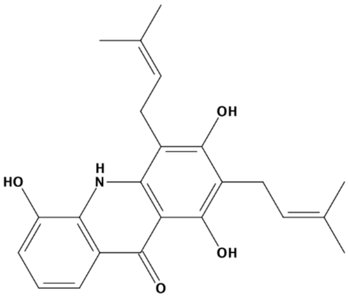

39
AnalytiCon Discovery
5JDI (-9.58 kcal/mol)

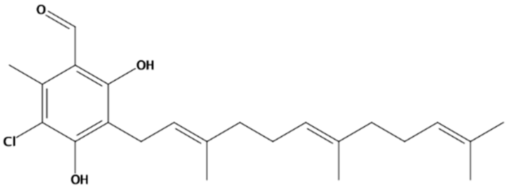

40
AnalytiCon Discovery
3QFX (-8.46 kcal/mol)

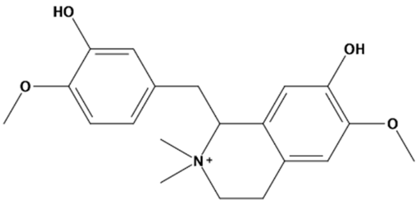

41
Specs NP
5JDI (-9.35 kcal/mol)

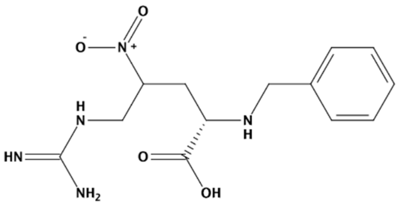

42
Specs NP
5JDI (-8.35 kcal/mol)

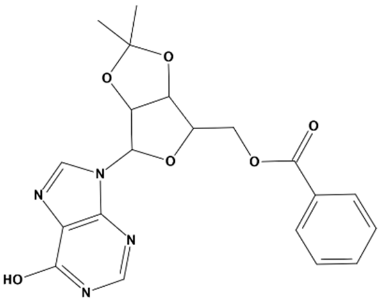

43
Specs NP
3MCV (-8.76 kcal/mol)
4CMK (-8.05 kcal/mol)

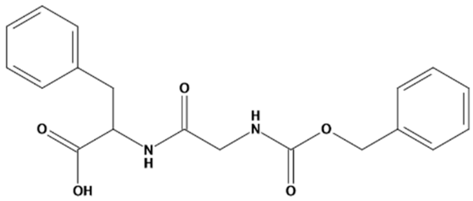

44
Specs NP
5JDI (-9.29 kcal/mol)

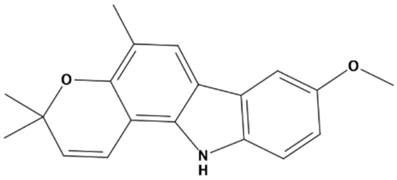

45
Specs NP
5JDI (-8.57 kcal/mol)

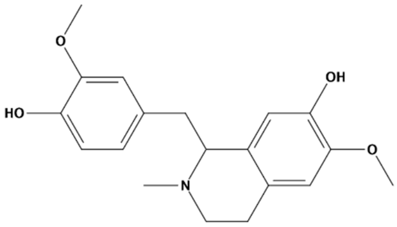

46
Specs NP
2X9G (-8.67 kcal/mol)

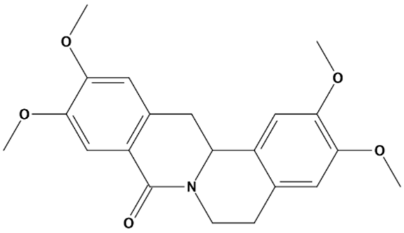

47
Specs NP
3MCV (-9.15 kcal/mol)

Figure S1. Molecular structures of the remaining *in silico* top hits that were tested *in vitro* against the target enzymes. The respective origin databases as well as protein structure models that were employed for their identification are listed.

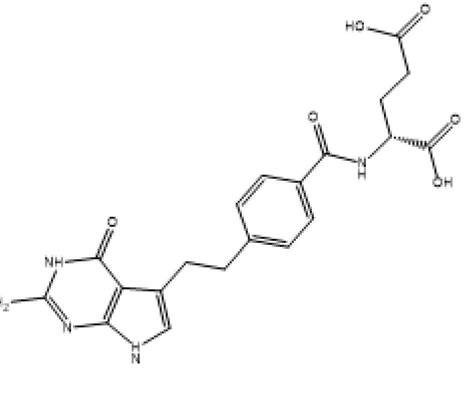

pemetrexed (2X9G)
-9.79 kcal/mol

Figure S2. Co-crystallized inhibitor pemetrexed of the *Tb*PTR1 protein structure model "2X9G". Complex- and target-based pharmacophore hypotheses based on the *Tb*PTR1 (ID: "2X9G") binding pocket can be found in the Supplementary Materials of our previous publication [4].

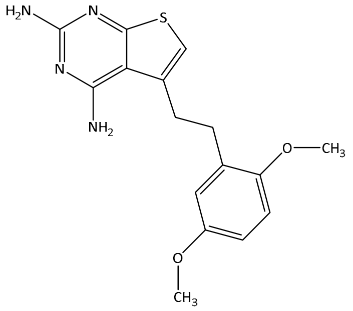

PY848 (3MCV)
-10.37 kcal/mol

Figure S3. Co-crystallized inhibitor PY848 of the *Tb*PTR1 protein structure model "3MCV". Complex- and target-based pharmacophore hypotheses based on the *Tb*PTR1 (ID: "3MCV") binding pocket can be found in the Supplementary Materials of our previous publication [4].

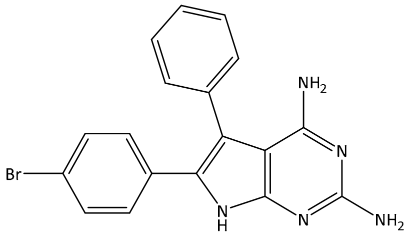

6-(4-bromophenyl)-5-phenyl-7H-pyrrolo[2,3-d]pyrimidine-2,4-diamine (4CMJ)
-9.51 kcal/mol

Figure S4. Co-crystallized inhibitor 6-(4-bromophenyl)-5-phenyl-7H-pyrrolo[2,3-d]pyrimidine-2,4-diamine of the *Tb*PTR1 protein structure model "4CMJ". Complex- and target-based pharmacophore hypotheses based on the *Tb*PTR1 (ID: "4CMJ") binding pocket can be found in the Supplementary Materials of our previous publication [4].

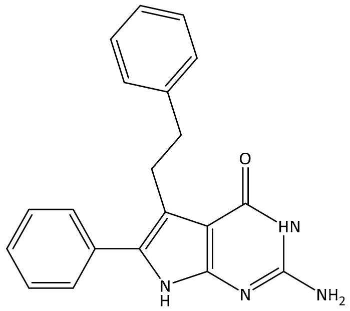

2-amino-5-phenethyl-6-phenyl-3H-pyrrolo[2,3 d]pyrimidine-4(7H)-one (4CMK)
-8.77 kcal/mol

Figure S5. Co-crystallized inhibitor 2-amino-5-phenethyl-6-phenyl-3H-pyrrolo[2,3 d]pyrimidine-4(7H)-one of the *Tb*PTR1 protein structure model "4CMK". Complex- and target-based pharmacophore hypotheses based on the *Tb*PTR1 (ID: "4CMK") binding pocket can be found in the Supplementary Materials of our previous publication [4].

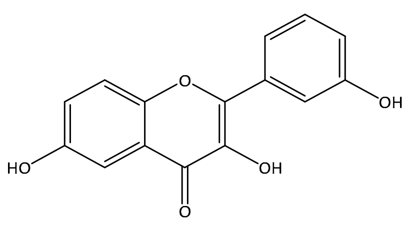

3,6-dihydroxy-2-(3 hydroxyphenyl)-4H-1-benzopyran-4-one (5JDI)
-7.65 kcal/mol

Figure S6. Co-crystallized inhibitor 3,6-dihydroxy-2-(3 hydroxyphenyl)-4H-1-benzopyran-4-one of the *Tb*PTR1 protein structure model "5JDI". Complex- and target-based pharmacophore hypotheses based on the *Tb*PTR1 (ID: "5JDI") binding pocket can be found in the Supplementary Materials of our previous publication [4].

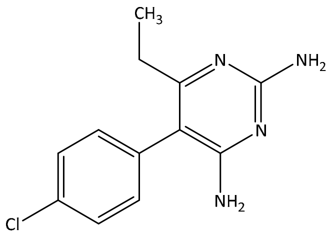

pyrimethamine (3QFX)
-8.26 kcal/mol

Figure S7. Co-crystallized inhibitor pyrimethamine of the *Tb*DHFR protein structure model "3QFX". Complex- and target-based pharmacophore hypotheses based on the *Tb*DHFR (ID: "3QFX") binding pocket can be found in the Supplementary Materials of our previous publication [4].

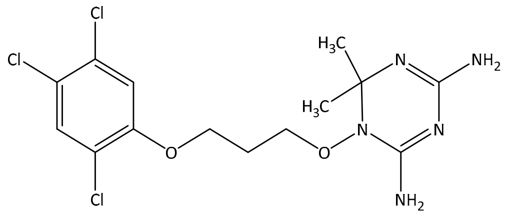

WR99210 (3RG9)
-8.66 kcal/mol

Figure S8. Co-crystallized inhibitor WR99210 of the *Tb*DHFR protein structure model "3RG9". Complex- and target-based pharmacophore hypotheses based on the *Tb*DHFR (ID: "3RG9") binding pocket can be found in the Supplementary Materials of our previous publication [4].

(**a**)

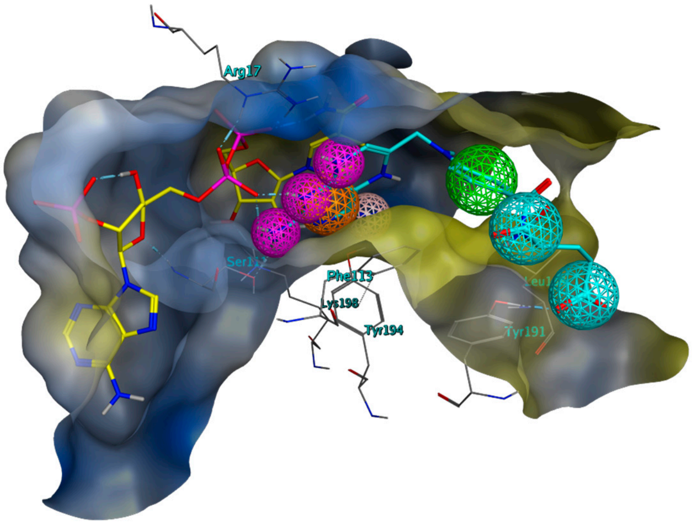

(**b**)

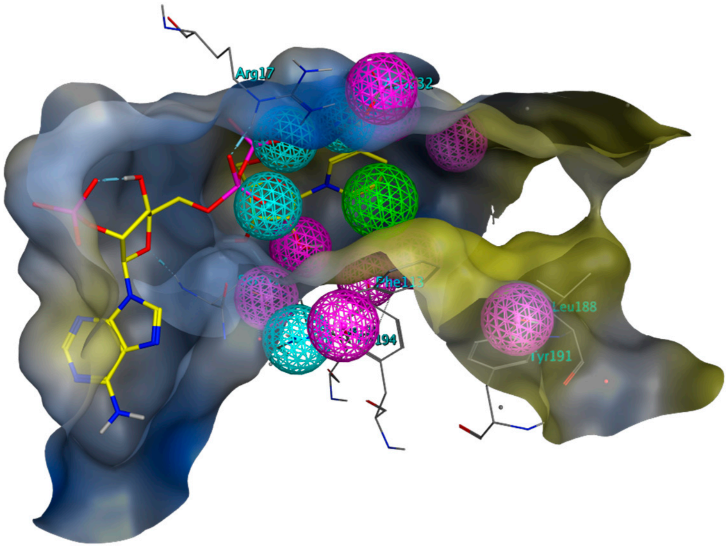

(**c**)

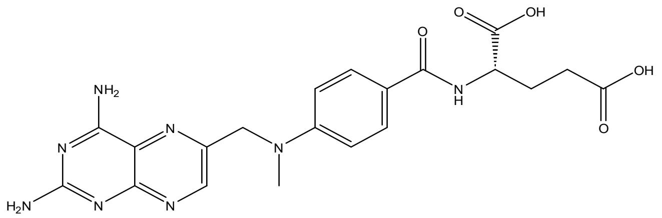

methotrexate (1E7W)
-10.59 kcal/mol

Figure S9. Complex- (a) and target-based (b) pharmacophore hypotheses based on the *Lm*PTR1 (ID: "1E7W") as well as the co-crystallized inhibitor methotrexate (c). Carbon atoms of the co-crystallized co-substrate NADP in yellow, carbon atoms of (**c**) in cyan. The molecular surface is colored according to lipophilicity with lipophilic areas in yellow and hydrophilic areas in blue. Potential interactions of the inhibitor are represented by feature spheres: H-bond donors in purple, H-bond acceptors in cyan, ionic interactions in beige, aromatic centers in orange, hydrophobic structures in green. Exclusion spheres are not depicted.

(**a**)

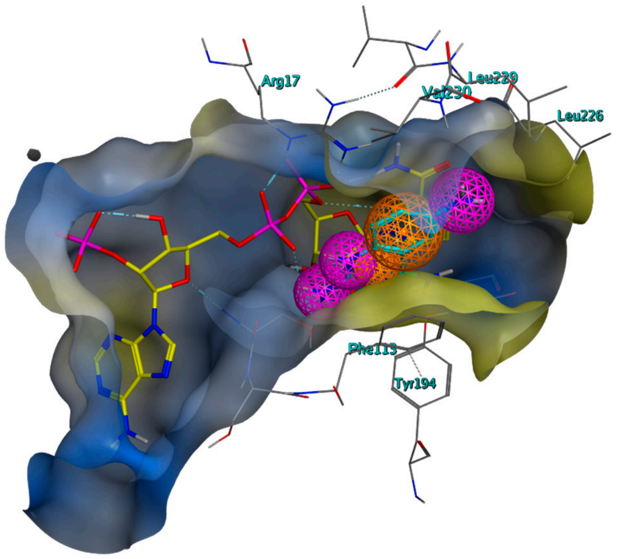

(**b**)

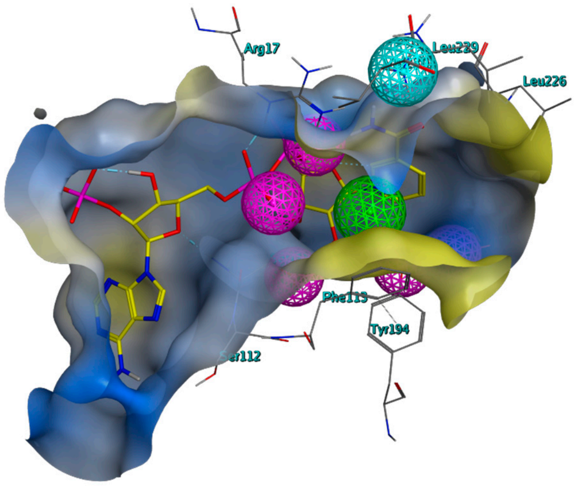

(**c**)

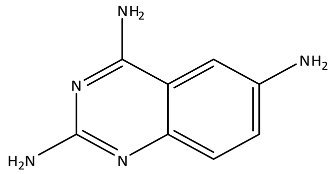

2,4,6-triaminoquinazoline (1W0C)
-7.05 kcal/mol

Figure S10. Complex- (a) and target-based (b) pharmacophore hypotheses based on the *Lm*PTR1 (ID: "1W0C") as well as the co-crystallized inhibitor 2,4,6-triaminoquinazoline (c). Carbon atoms of the co-crystallized co-substrate NADP in yellow, carbon atoms of (**c**) in cyan. The molecular surface is colored according to lipophilicity with lipophilic areas in yellow and hydrophilic areas in blue. Potential interactions of the inhibitor are represented by feature spheres: H-bond donors in purple, H-bond acceptors in cyan, ionic interactions in beige, aromatic centers in orange, hydrophobic structures in green. Exclusion spheres are not depicted.

(**a**)

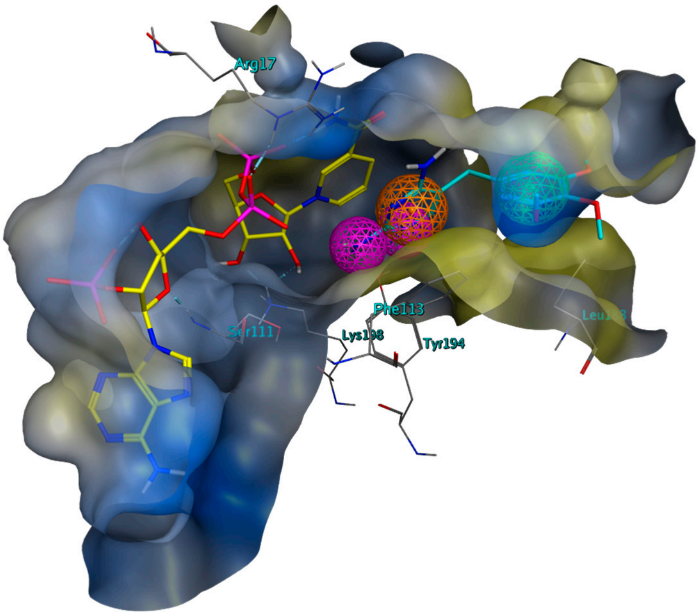

(**b**)

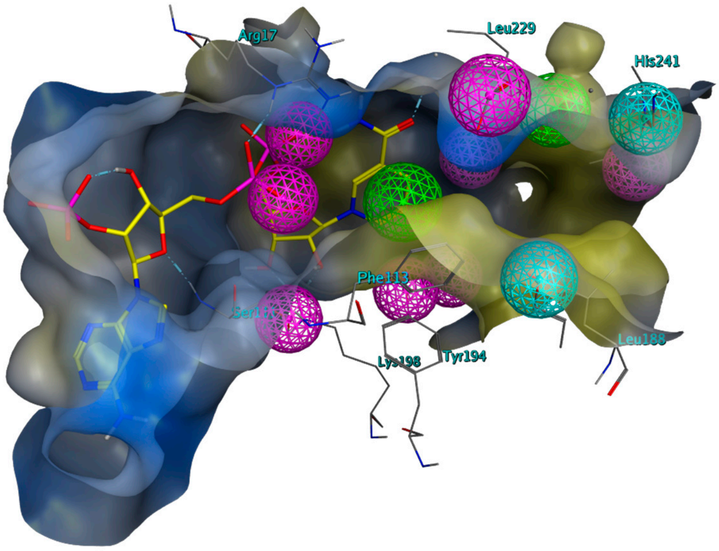

(**c**)

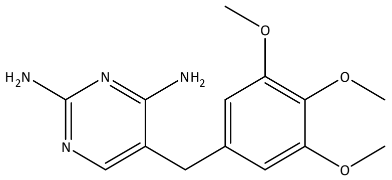

trimethoprim (2BFM)
-7.76 kcal/mol

Figure S11. Complex- (a) and target-based (b) pharmacophore hypotheses based on the *Lm*PTR1 (ID: "2BFM") as well as the co-crystallized inhibitor trimethoprim (c). Carbon atoms of the co-crystallized co-substrate NADP in yellow, carbon atoms of (**c**) in cyan. The molecular surface is colored according to lipophilicity with lipophilic areas in yellow and hydrophilic areas in blue. Potential interactions of the inhibitor are represented by feature

spheres: H-bond donors in purple, H-bond acceptors in cyan, aromatic centers in orange, hydrophobic structures in green. Exclusion spheres are not depicted.

(**a**)

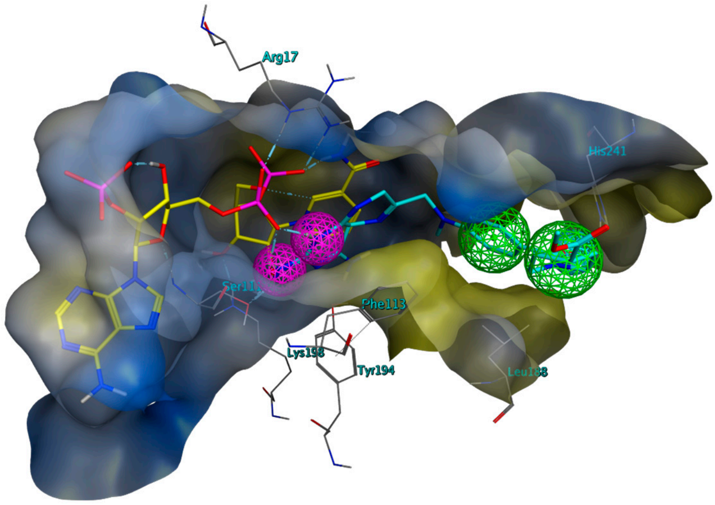

(**b**)

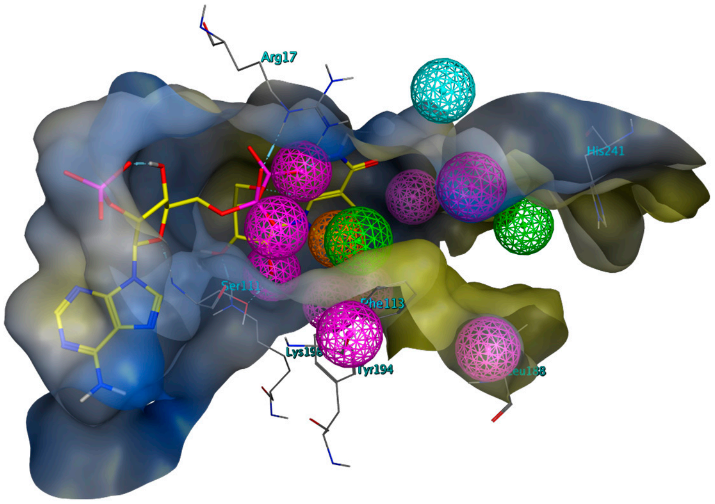

(**c**)

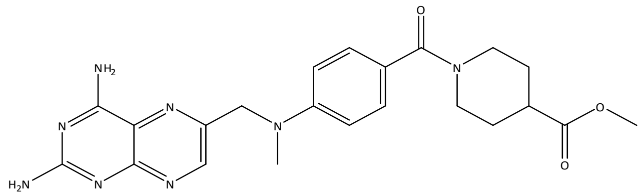

methyl-1-(4-{[(2,4-diaminopteridin-6-yl)methyl](methyl)amino}benzoyl)piperidine-4-carboxylate
(2QHX)
-9.34 kcal/mol

Figure S12. Complex- (a) and target-based (b) pharmacophore hypotheses based on the *Lm*PTR1 (ID: "2QHX") as well as the co-crystallized inhibitor methyl-1-(4-{[(2,4-diaminopteridin-6-yl)methyl](methyl)amino}benzoyl)piperidine-4-carboxylate (c). Carbon atoms of the co-crystallized co-substrate NADP in yellow, carbon atoms of (**c**) in cyan. The molecular surface is colored according to lipophilicity with lipophilic areas in yellow and hydrophilic areas in blue. Potential interactions of the inhibitor are represented by feature spheres: H-bond donors in purple, H-bond acceptors in cyan,

aromatic centers in orange, hydrophobic structures in green. Exclusion spheres are not depicted.

(**a**)

```
Pairwise Identity Matrix:
       The table value at row i, column j equals the number of residue
       matches between sequences i and j, divided by the length of
       sequence j.

    Chains      1     4
 1:3KJS.A          64.4
 4:LmDHFR    66.5


3KJS.A    ....XSLFKIRMPETVAEGTRLALRAFSLVVAVDERGGIGDGRSIPWNVPEDMKFFRDVT
LmDHFR    MSRAAARFKIPMPETKADFAFPSLRAFSIVVALDMQHGIGDGESIPWRVPEDMTFFKNQT

3KJS.A    TKLRGKNVKPSPAKRNAVVMGRKTWDSIPPKFRPLPGRLNVVLSSTLTTQHL........
LmDHFR    TLLRNKK.PPTEKKRNAVVMGRKTWESVPVKFRPLKGRLNIVLSSKATVEELLAPLPEGQ

3KJS.A    ...XXDSIVAVNGGLEQALQLLASPNYTPSIETVYCIGGGSVYAEALRPPCVHLLQAIYR
LmDHFR    RAAAAQDVVVVNGGLAEALRLLARPLYCSSIETAYCVGGAQVYADAMLSPCIEKLQEVYL

3KJS.A    TTIRASESSCSVFFRVPESGTEAAAGIEWQRETISEELTSANGNETKYYFEKLIPRNREE
LmDHFR    TRIYATAPACTRFFPFPPENAATA....WDLAS.SQGRRKSEAEGLEFEICKYVPRNHEE

3KJS.A    EQYLSLVDRIIREGNVKHDRTGVGTLSIFGAQMRFSLRNNRLPLLTTKRVFWRGVCEELL
LmDHFR    RQYLELIDRIMKTGIVKEDRTGVGTISLFGAQMRFSLRDNRLPLLTTKRVFWRGVCEELL

3KJS.A    WFLRGETYAKKLSDKGVHIWDDNGSRAFLDSRGLTEYEEMDLGPVYGFQWRHFGAAYTHH
LmDHFR    WFLRGETSAQLLADKDIHIWDGNGSREFLDSRGLTENKEMDLGPVYGFQWRHFGADYKGF

3KJS.A    DANYDGQGVDQIKAIVETLKTNPDDRRMLFTAWNPSALPRMALPPCHLLAQFYVSN..GE
LmDHFR    EANYDGEGVDQIKLIVETIKTNPNDRRLLVTAWNPCALQKMALPPCHLLAQFYVNTDTSE

3KJS.A    LSCMLYQRSCDMGLGVPFNIASYALLTILIAKATGLRPGELVHTLGDAHVYSNHVEPCNE
LmDHFR    LSCMLYQRSCDMGLGVPFNIASYALLTILIAKATGLRPGELVHTLGDAHVYRNHVDALKA

3KJS.A    QLKRVPRAFPYLVFRREREFLEDYEEGDMEVIDYAPYPPIX.....
LmDHFR    QLERVPHAFPTLIFKEERQYLEDYELTDMEVIDYVPHPAIKMEMAV
```

(**b**)

```
Pairwise Similarity Matrix:
       The table value at row i, column j equals the number of positive
       matches between sequences i and j, divided by the length of
       sequence j. A residue substitution is positive if the BLOSUM62
       substitution score is greater than zero

    Chains      1     4
 1:3KJS.A          74.8
 4:LmDHFR    77.2


3KJS.A    ....XSLFKIRMPETVAEGTRLALRAFSLVVAVDERGGIGDGRSIPWNVPEDMKFFRDVT
LmDHFR    MSRAAARFKIPMPETKADFAFPSLRAFSIVVALDMQHGIGDGESIPWRVPEDMTFFKNQT

3KJS.A    TKLRGKNVKPSPAKRNAVVMGRKTWDSIPPKFRPLPGRLNVVLSSTLTTQHL........
LmDHFR    TLLRNKK.PPTEKKRNAVVMGRKTWESVPVKFRPLKGRLNIVLSSKATVEELLAPLPEGQ

3KJS.A    ...XXDSIVAVNGGLEQALQLLASPNYTPSIETVYCIGGGSVYAEALRPPCVHLLQAIYR
LmDHFR    RAAAAQDVVVVNGGLAEALRLLARPLYCSSIETAYCVGGAQVYADAMLSPCIEKLQEVYL

3KJS.A    TTIRASESSCSVFFRVPESGTEAAAGIEWQRETISEELTSANGNETKYYFEKLIPRNREE
LmDHFR    TRIYATAPACTRFFPFPPENAATA....WDLAS.SQGRRKSEAEGLEFEICKYVPRNHEE

3KJS.A    EQYLSLVDRIIREGNVKHDRTGVGTLSIFGAQMRFSLRNNRLPLLTTKRVFWRGVCEELL
LmDHFR    RQYLELIDRIMKTGIVKEDRTGVGTISLFGAQMRFSLRDNRLPLLTTKRVFWRGVCEELL

3KJS.A    WFLRGETYAKKLSDKGVHIWDDNGSRAFLDSRGLTEYEEMDLGPVYGFQWRHFGAAYTHH
LmDHFR    WFLRGETSAQLLADKDIHIWDGNGSREFLDSRGLTENKEMDLGPVYGFQWRHFGADYKGF

3KJS.A    DANYDGQGVDQIKAIVETLKTNPDDRRMLFTAWNPSALPRMALPPCHLLAQFYVSN..GE
LmDHFR    EANYDGEGVDQIKLIVETIKTNPNDRRLLVTAWNPCALQKMALPPCHLLAQFYVNTDTSE

3KJS.A    LSCMLYQRSCDMGLGVPFNIASYALLTILIAKATGLRPGELVHTLGDAHVYSNHVEPCNE
LmDHFR    LSCMLYQRSCDMGLGVPFNIASYALLTILIAKATGLRPGELVHTLGDAHVYRNHVDALKA

3KJS.A    QLKRVPRAFPYLVFRREREFLEDYEEGDMEVIDYAPYPPIX.....
LmDHFR    QLERVPHAFPTLIFKEERQYLEDYELTDMEVIDYVPHPAIKMEMAV
```

Figure S13. Sequence alignment of the *Tc*DHFR-TS chain A ("3KJS.A") and the *Lm*DHFR-TS ("*Lm*DHFR"). Percent sequence identity (**a**) and similarity (**b**) is calculated by dividing the number of identical amino acids between the two chains by the total number of amino acids. The relative

sequence similarity and sequence identity compared to the template structure amounted to 76.8 % and 66.3 %, respectively.

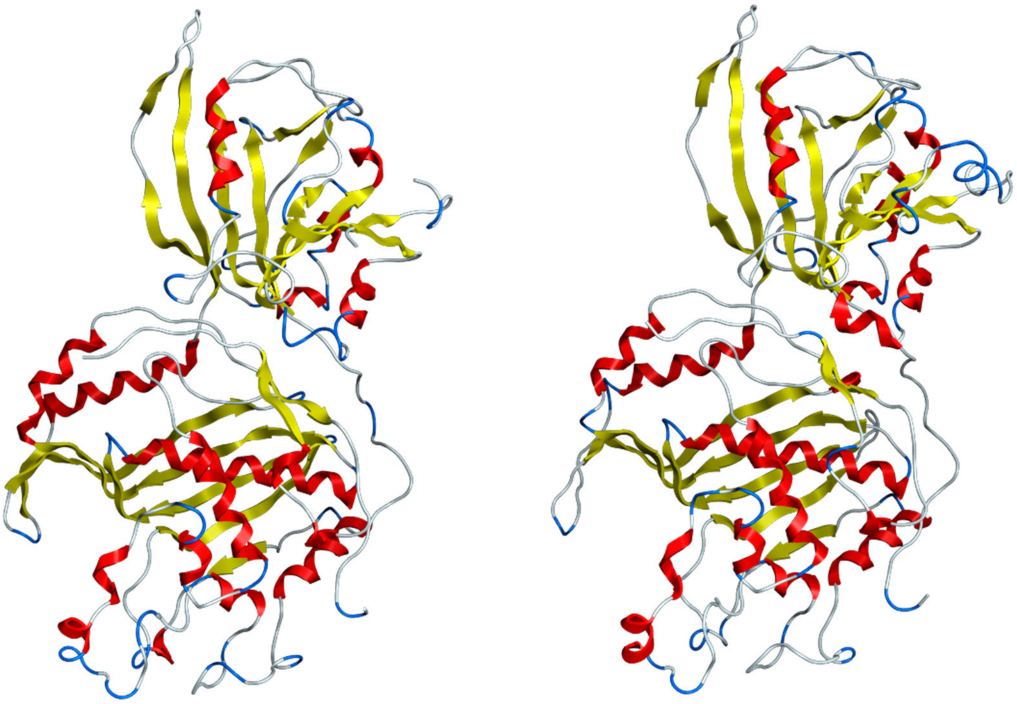

(**a**) (**b**)

Figure S14. Ribbon diagram of the secondary structural elements of the *Tc*DHFR-TS template "3KJS.A" (a) and the homology model of *Lm*DHFR-TS (b). The secondary structures are color-coded (ribbon loop: white; α-, π-, 3_{10}-helix: red; strand: yellow; turn: blue).

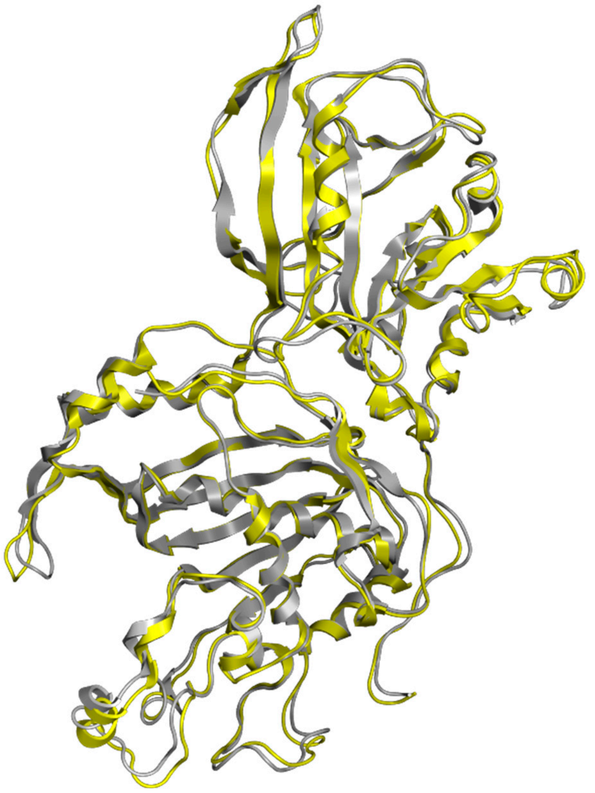

Figure S15. Structural superposition of the *Tc*DHFR-TS template "3KJS.A" (gray) and the homology model of *Lm*DHFR-TS (yellow) as ribbon diagram.

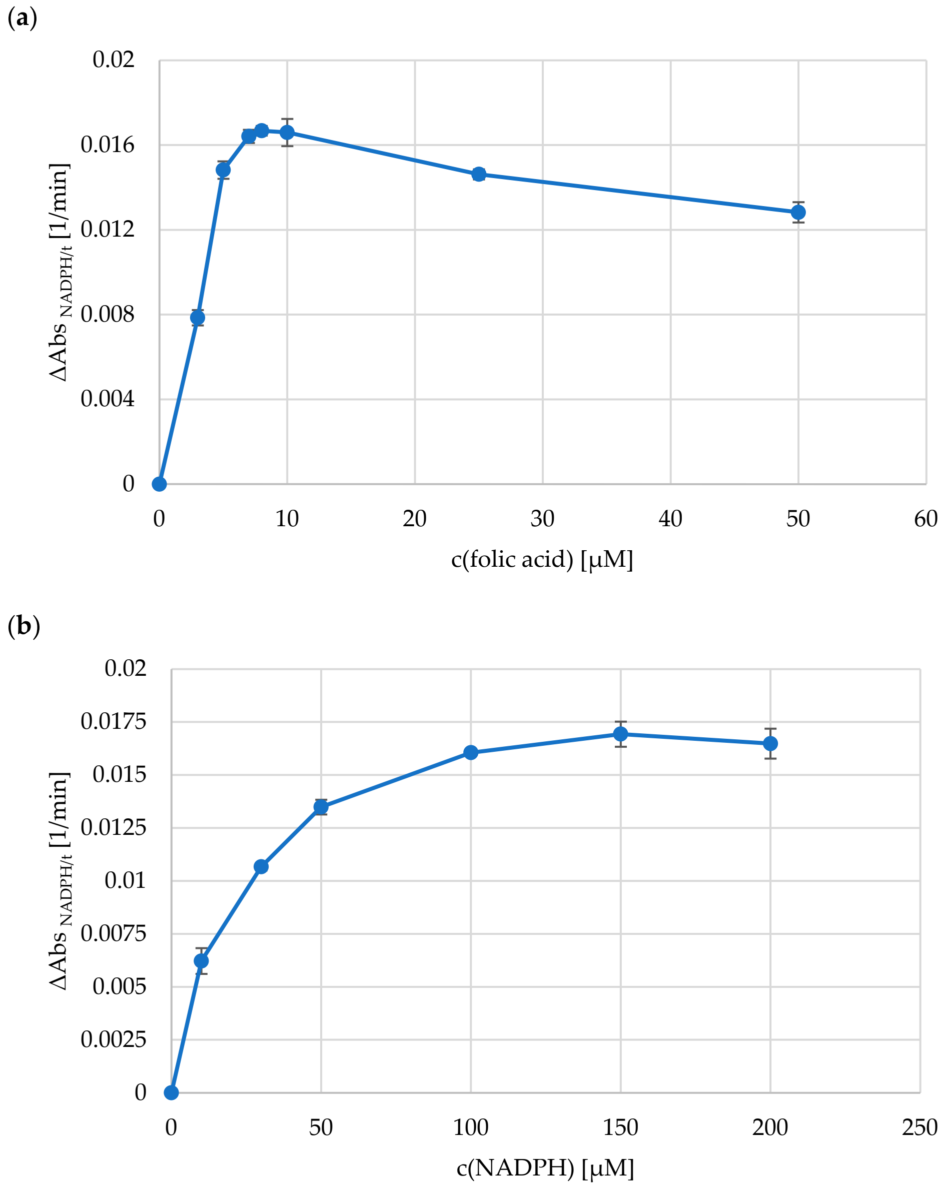

Figure S16. Experimental determination of the saturating conditions of folic acid and NADPH for *Tb*PTR1. (**a**) Constant concentration of the co-substrate NADPH (200 µM) while varying the concentrations of the substrate folic acid (3 µM – 50 µM). In the concentration range above the saturation (8-10 µM), substrate inhibition was observed. (**b**) Constant concentration of the substrate folic acid (8 µM) while varying the concentrations of the co-substrate NADPH (10 µM – 200 µM). The determination was carried out according to 4.2.4, using buffer A (50 mM Tris/HCl (pH 7.6), 250 mM NaCl) at 340 nm and a constant temperature of 30 °C.

(**a**)

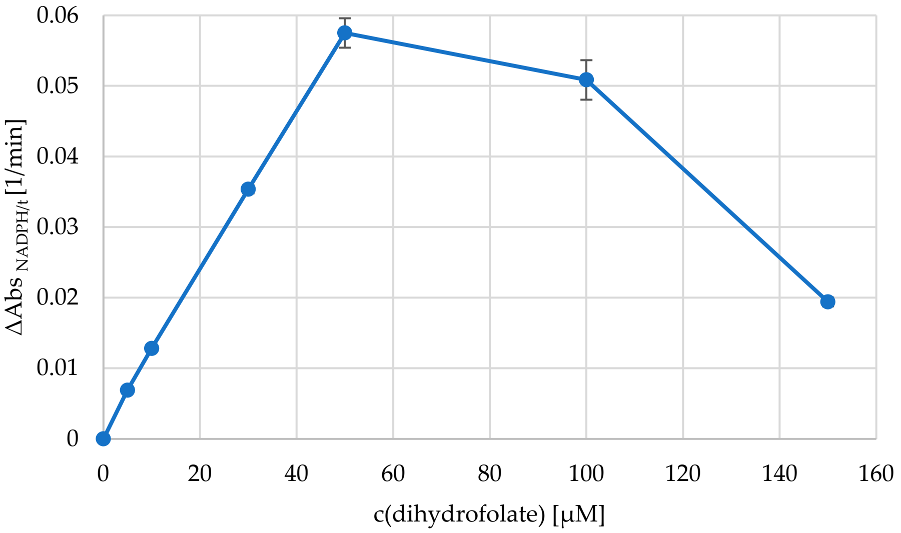

(**b**)

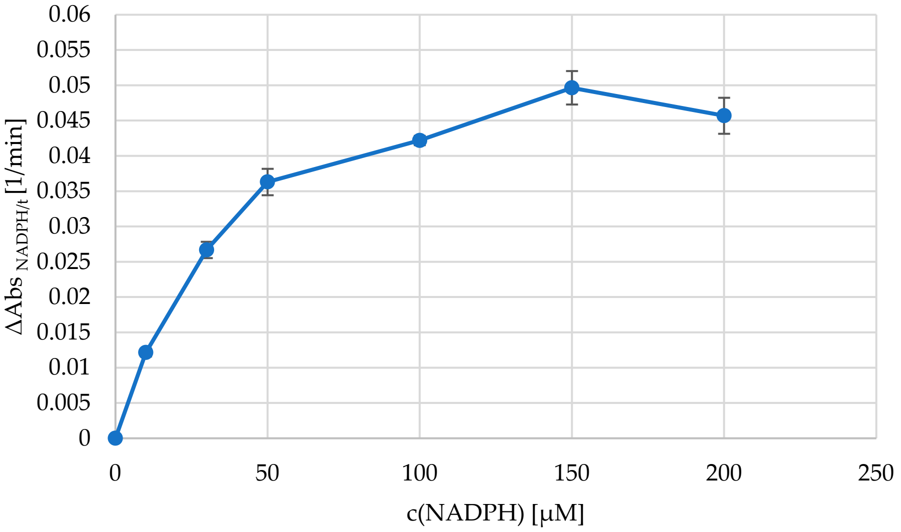

Figure S17. Experimental determination of the saturating conditions of dihydrofolate (DHF) and NADPH for *Tb*DHFR. (**a**) Constant concentration of the co-substrate NADPH (200 µM) while varying the concentrations of the substrate DHF (5 µM – 150 µM). In the concentration range above saturation (>40 µM), substrate inhibition was observed. (**b**) Constant concentration of the substrate DHF (50 µM) while varying the concentrations of the co-substrate NADPH (10 µM – 200 µM). The determination was carried out according to 4.2.4, using buffer C (50 mM Tris/HCl (pH 7.6), 250 mM NaCl, 10 mM BME) at 340 nm and a constant temperature of 30 °C.

(**a**)

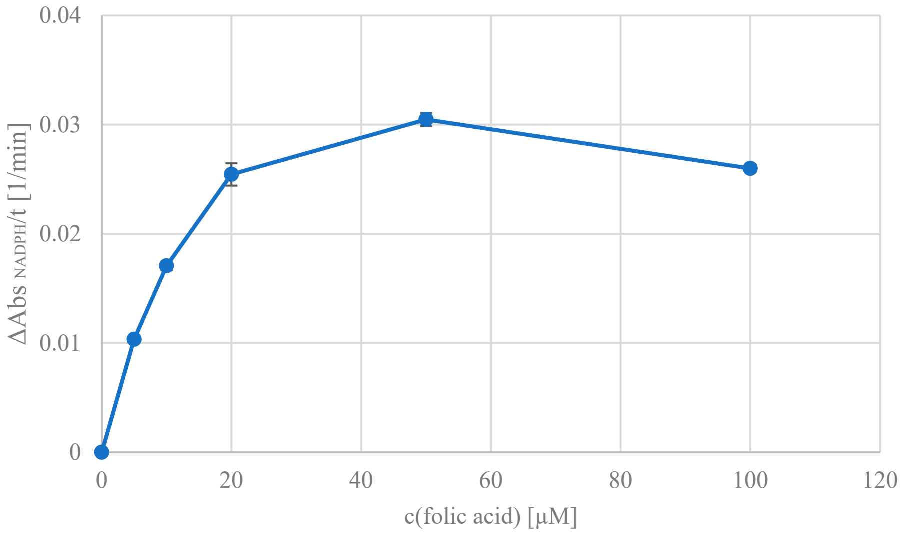

(**b**)

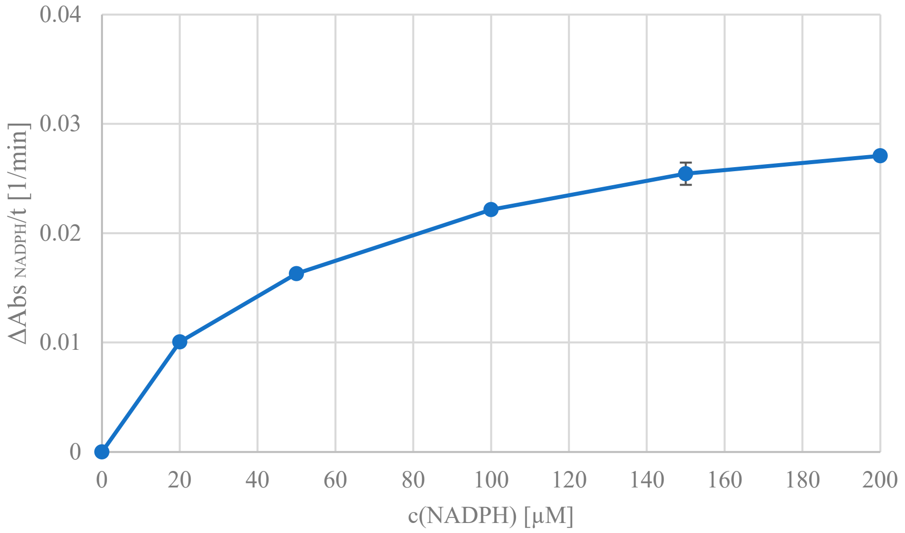

Figure S18. Experimental determination of the saturating conditions of folic acid and NADPH for *Lm*PTR1. (**a**) Constant concentration of the co-substrate NADPH (200 µM) while varying the concentrations of the substrate folic acid (5 µM – 100 µM). In the concentration range above saturation (>50 µM), substrate inhibition was observed. (**b**) Constant concentration of the substrate folic acid (50 µM) while varying the concentrations of the co-substrate NADPH (20 µM – 200 µM). The determination was carried out according to 4.2.4, using buffer B (50 mM NaH_2PO_4 (pH 6.0), 100 mM NaCl) at 340 nm and a constant temperature of 30 °C.

(**a**)

(**b**)

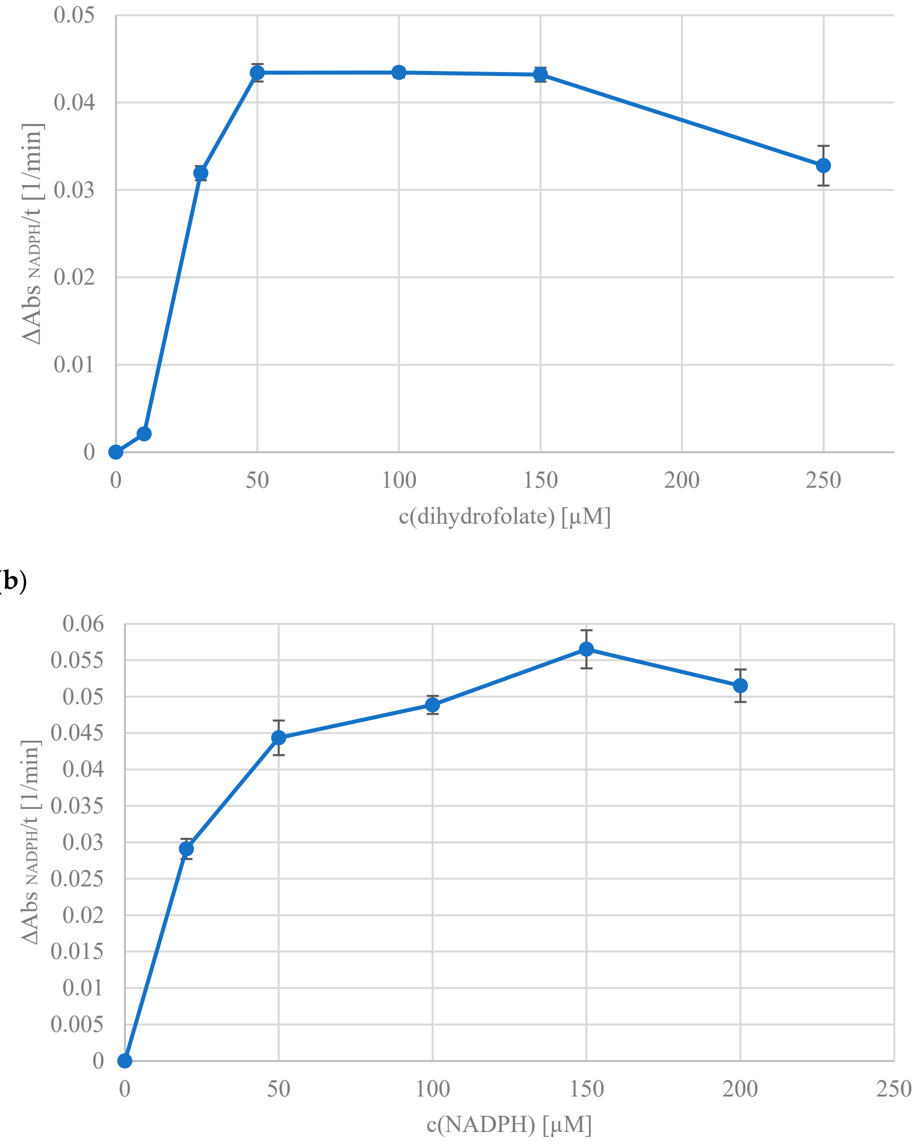

Figure S19. Experimental determination of the saturating conditions of dihydrofolate (DHF) and NADPH for *Lm*DHFR. (**a**) Constant concentration of the co-substrate NADPH (150 µM) while varying the concentrations of the substrate DHF (10 µM – 250 µM). In the concentration range above saturation (>100 µM), substrate inhibition was observed. (**b**) Constant concentration of the substrate DHF (50 µM) while varying the concentrations of the co-substrate NADPH (20 µM – 200 µM). The determination was carried out according to 4.2.4, using buffer C (50 mM Tris/HCl (pH 7.6), 250 mM NaCl, 10 mM BME) at 340 nm and a constant temperature of 30 °C.

(**a**)

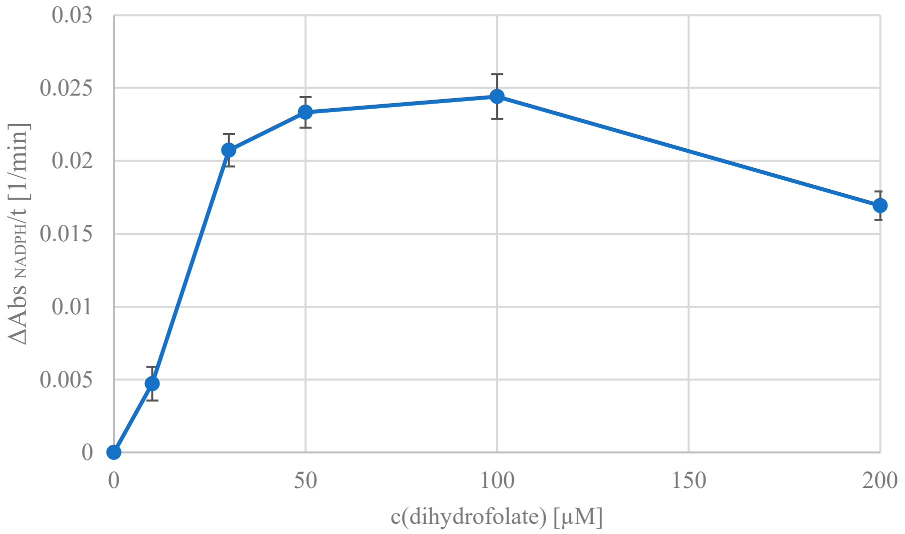

(**b**)

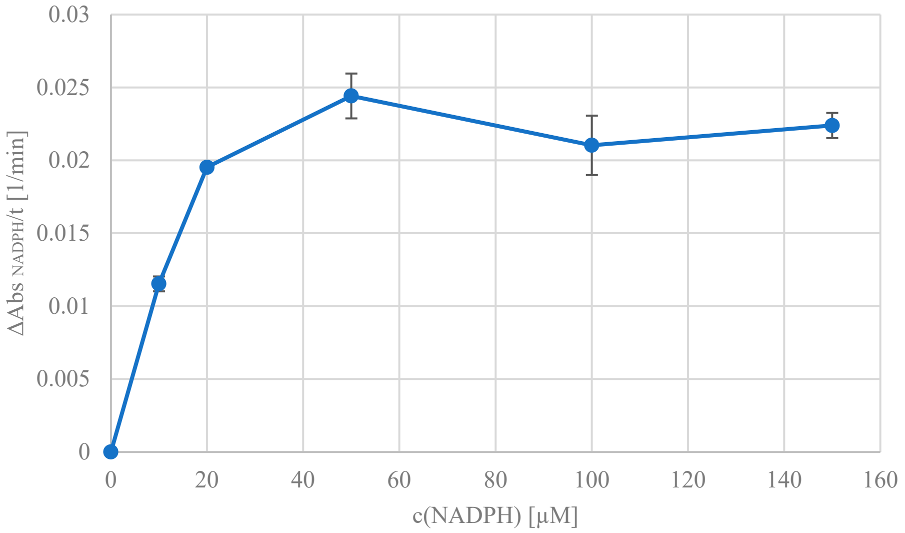

Figure S20. Experimental determination of the saturating conditions of dihydrofolate (DHF) and NADPH for *h*DHFR. (**a**) Constant concentration of the co-substrate NADPH (150 µM) while varying the concentrations of the substrate DHF (10 µM – 200 µM). In the concentration range above saturation (>100 µM), substrate inhibition was observed. (**b**) Constant concentration of the substrate DHF (100 µM) while varying the concentrations of the co-substrate NADPH (10 µM – 150 µM). The determination was carried out according to 4.2.4, using buffer C (50 mM Tris/HCl (pH 7.6), 250 mM NaCl, 10 mM BME) at 340 nm and a constant temperature of 30 °C.

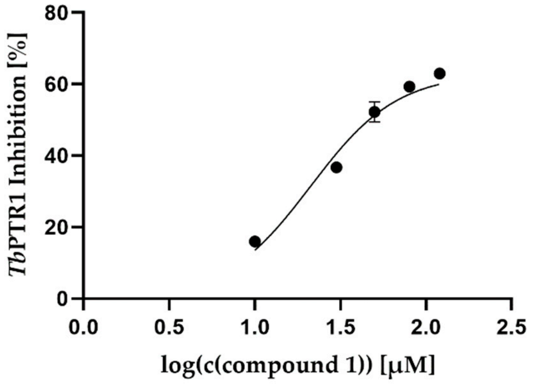

Figure S21. Determination of the IC_{50}/EC_{50} value of compound **1** against *Tb*PTR1 through nonlinear regression analysis using the software GraphPad Prism 8 (Table 1).

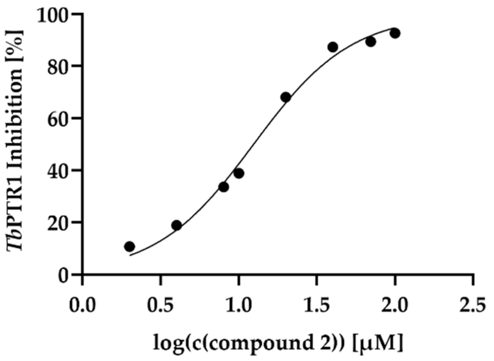

(**a**)

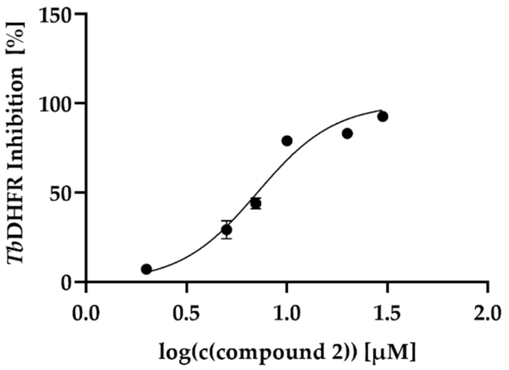

(**b**)

Figure S22. Determination of the IC_{50}/EC_{50} values of compound **2** against *Tb*PTR1 (**a**) and *Tb*DHFR (**b**) through nonlinear regression analysis using the software GraphPad Prism 8 (Table 1).

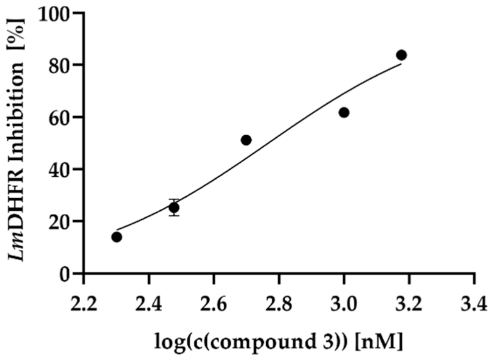

(**a**)

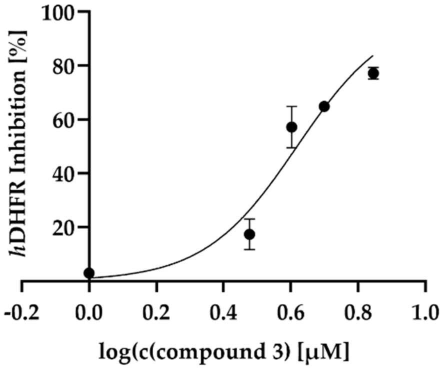

(**b**)

Figure S23. Determination of the IC_{50}/EC_{50} values of compound **3** against *Lm*DHFR (**a**) and *h*DHFR (**b**) through nonlinear regression analysis using the software GraphPad Prism 8 (Table 1).

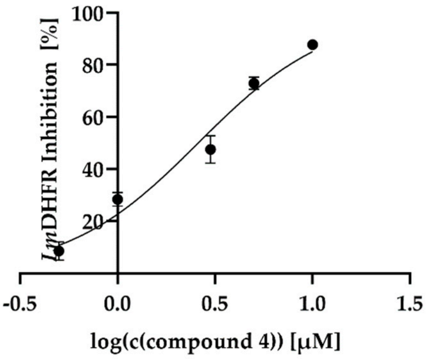

(**a**)

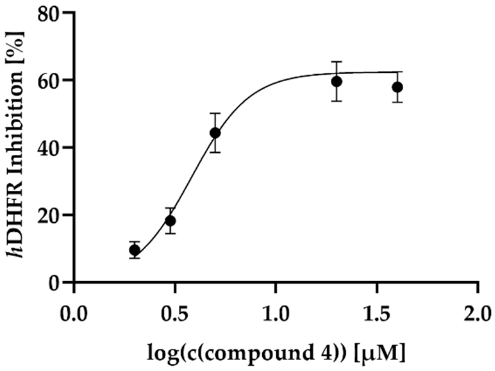

(**b**)

Figure S24. Determination of the IC_{50}/EC_{50} values of compound **4** against *Lm*DHFR (**a**) and *h*DHFR (**b**) through nonlinear regression analysis using the software GraphPad Prism 8 (Table 1).

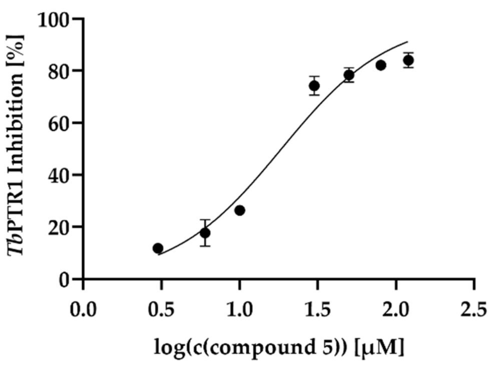

(**a**)

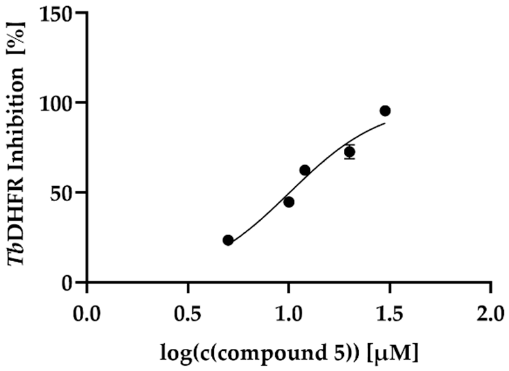

(**b**)

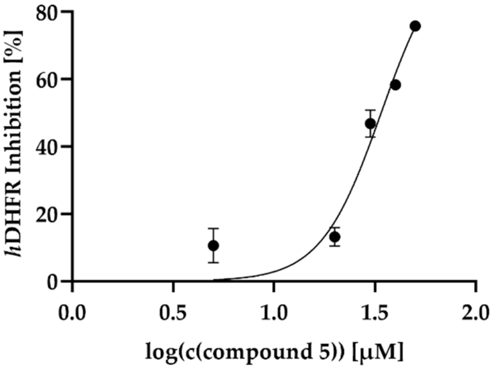

(**c**)

Figure S25. Determination of the IC_{50}/EC_{50} values of compound **5** against *Tb*PTR1 (**a**), *Tb*DHFR (**b**) and *h*DHFR (**c**) through nonlinear regression analysis using the software GraphPad Prism 8 (Table 1).

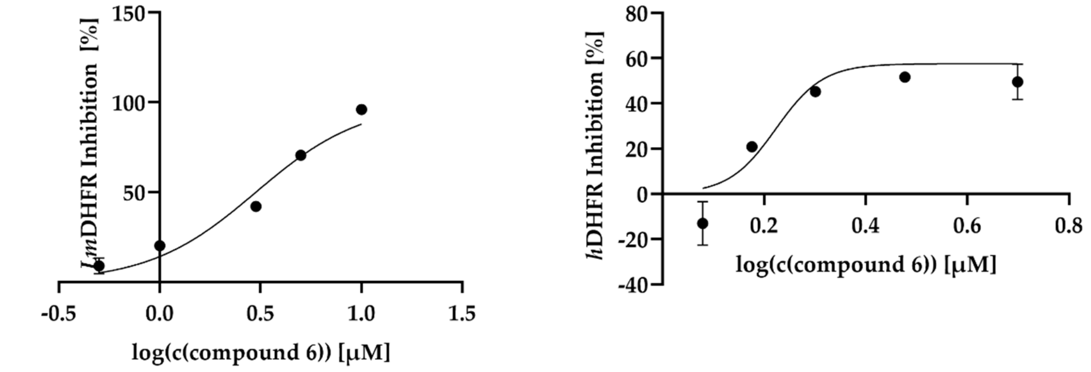

(**a**) (**b**)

Figure S26. Determination of the IC_{50}/EC_{50} values of compound **6** against *Lm*DHFR (**a**) and *h*DHFR (**b**) through nonlinear regression analysis using the software GraphPad Prism 8 (Table 1).

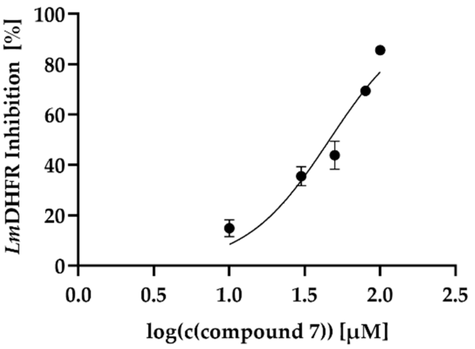

Figure S27. Determination of the IC_{50}/EC_{50} value of compound **7** against *Lm*DHFR through nonlinear regression analysis using the software GraphPad Prism 8 (Table 1).

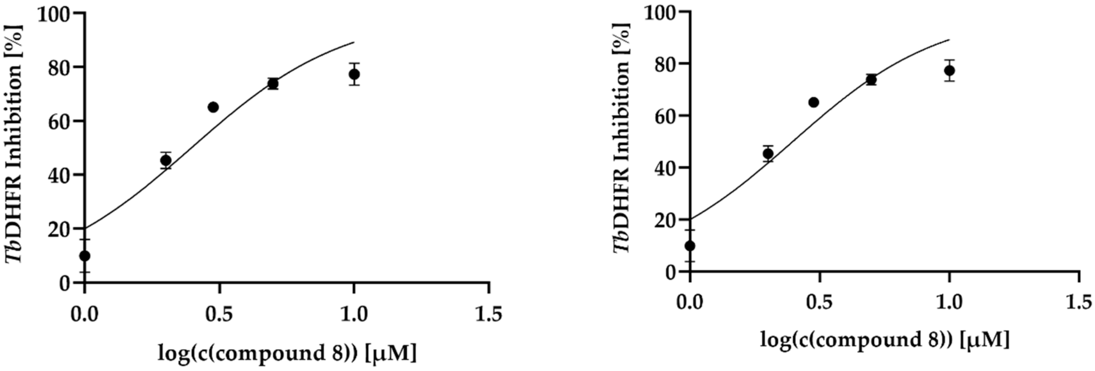

(**a**) (**b**)

Figure S28. Determination of the IC_{50}/EC_{50} value of compound **8** against *Tb*DHFR (**a**) and *Lm*DHFR (**b**) through nonlinear regression analysis using the software GraphPad Prism 8 (Table 1).

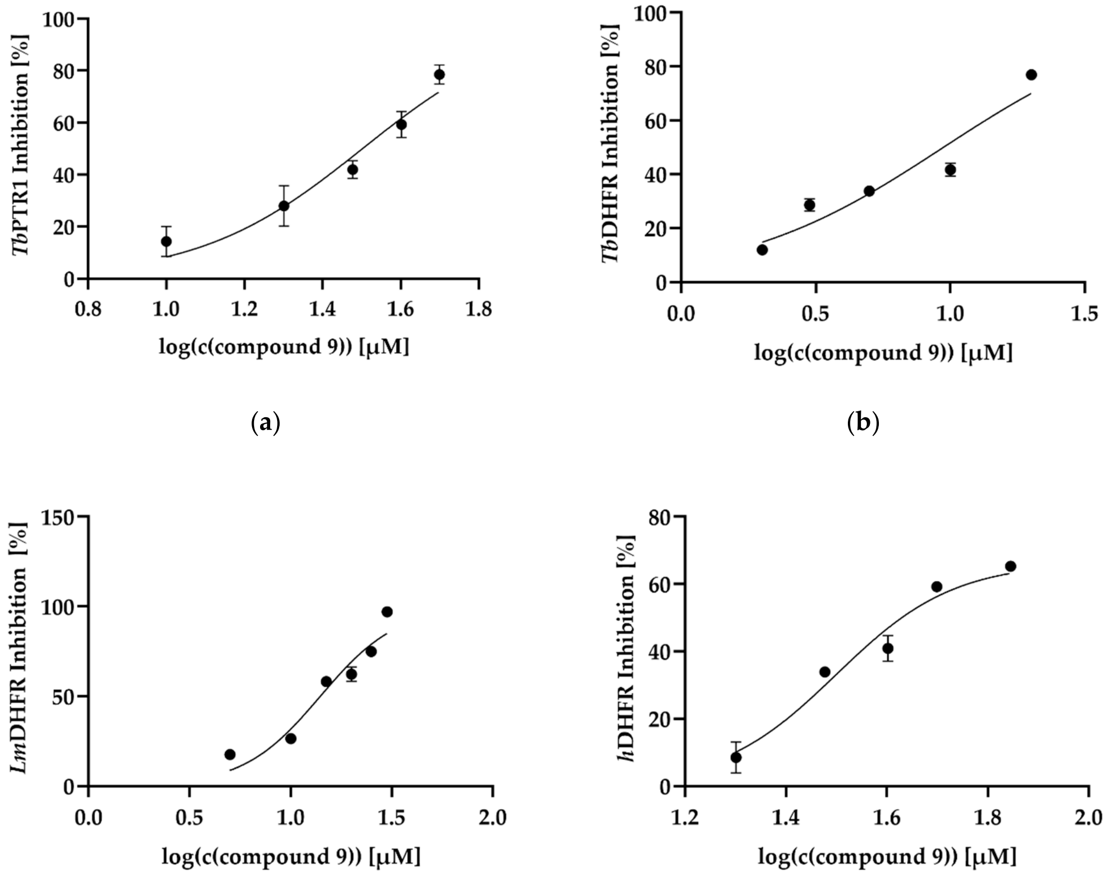

(**c**) (**d**)

Figure S29. Determination of the IC_{50}/EC_{50} values of compound **9** against *Tb*PTR1 (**a**), *Tb*DHFR (**b**), *Lm*DHFR (**c**) and *h*DHFR (**d**) through nonlinear regression analysis using the software GraphPad Prism 8 (Table 1).

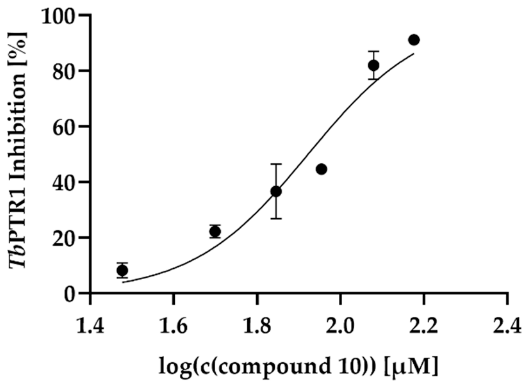

Figure S30. Determination of the IC_{50}/EC_{50} value of compound **10** against *Tb*PTR1 through nonlinear regression analysis using the software GraphPad Prism 8 (Table 1).

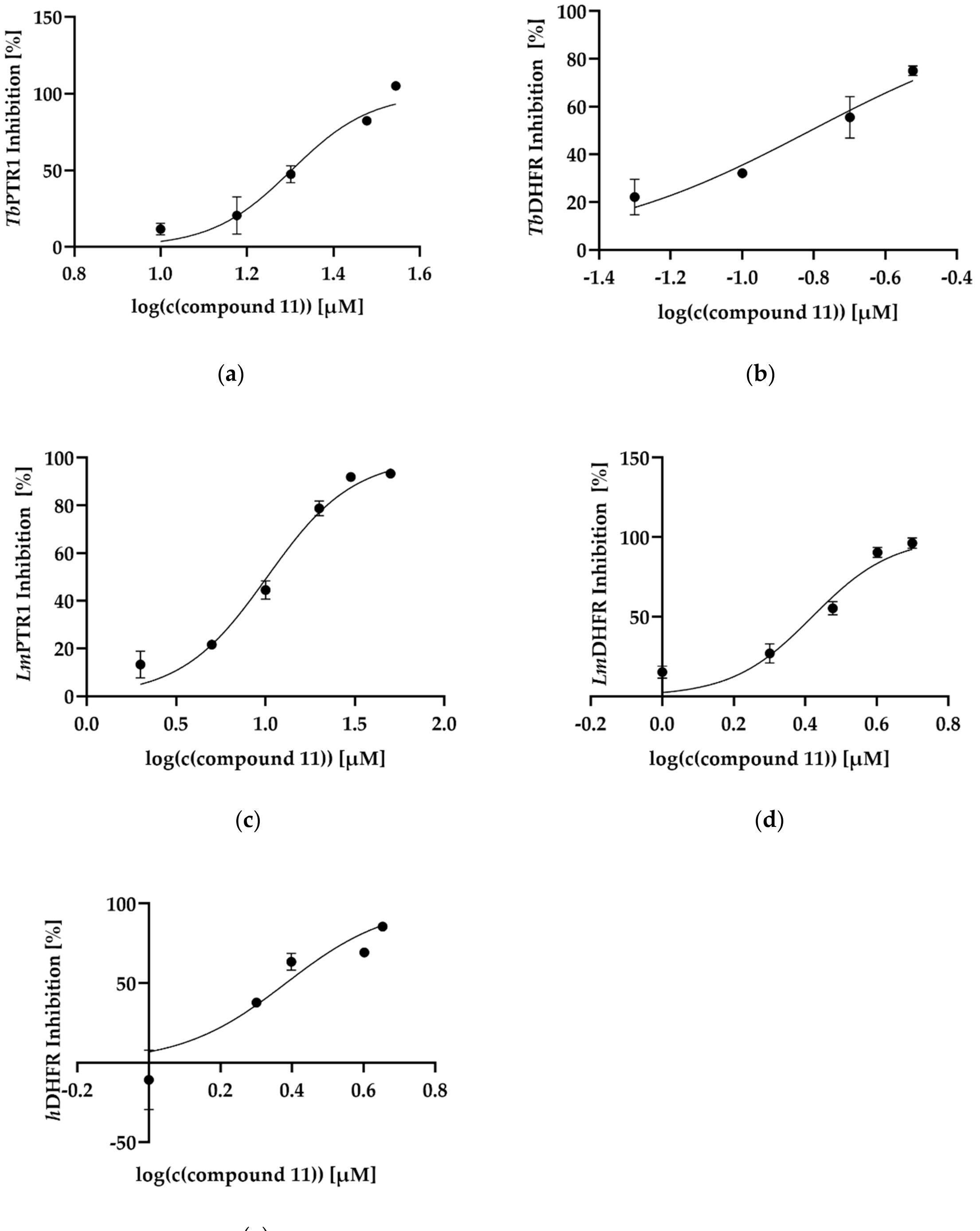

Figure S31. Determination of the IC_{50}/EC_{50} values of compound **11** against *Tb*PTR1 (**a**), *Tb*DHFR (**b**), *Lm*PTR1 (**c**), *Lm*DHFR (**d**) and *h*DHFR (**e**) through nonlinear regression analysis using the software GraphPad Prism 8 (Table 1).

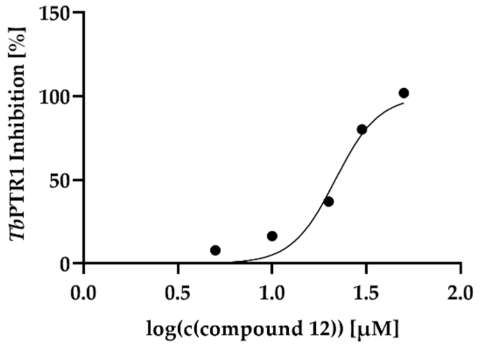

Figure S32. Determination of the IC_{50}/EC_{50} value of compound **12** against *Tb*PTR1 through nonlinear regression analysis using the software GraphPad Prism 8 (Table 1).

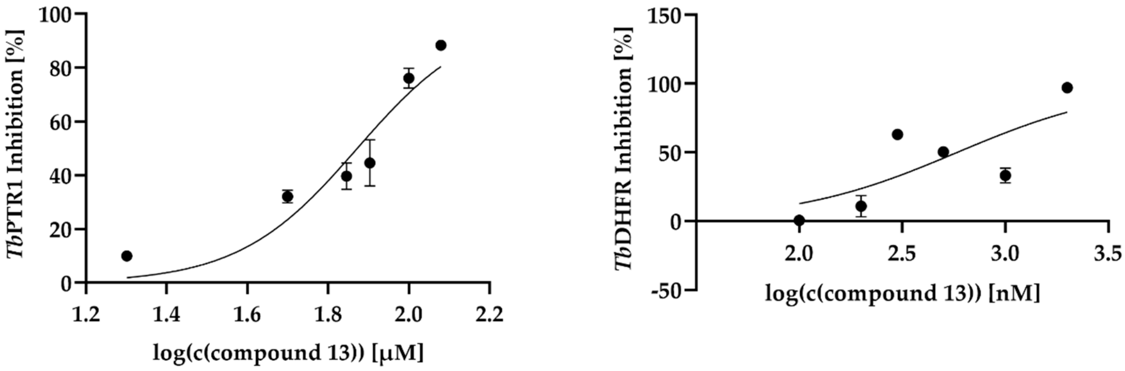

(**a**) (**b**)

Figure S33. Determination of the IC_{50}/EC_{50} values of compound **13** against *Tb*PTR1 (**a**) and *Tb*DHFR (**b**) through nonlinear regression analysis using the software GraphPad Prism 8 (Table 1).

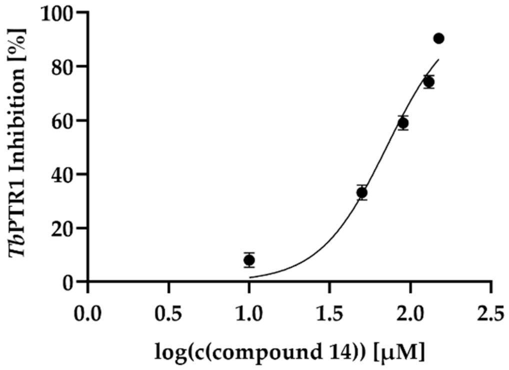

(**a**)

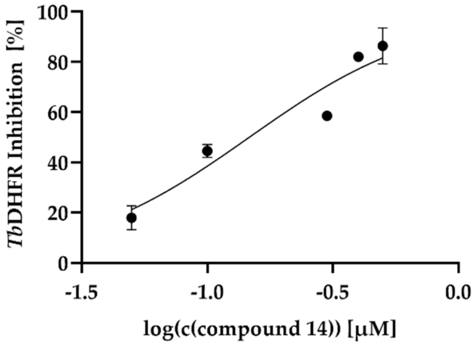

(**b**)

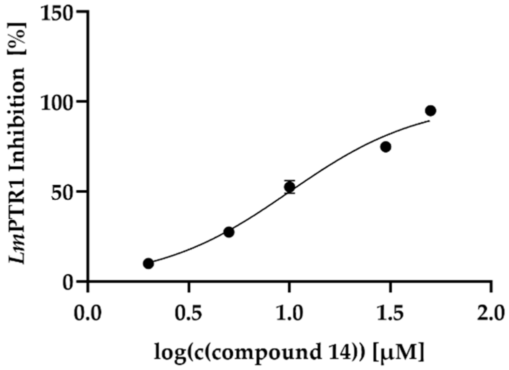

(**c**)

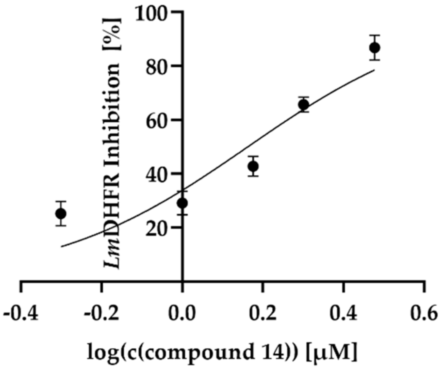

(**d**)

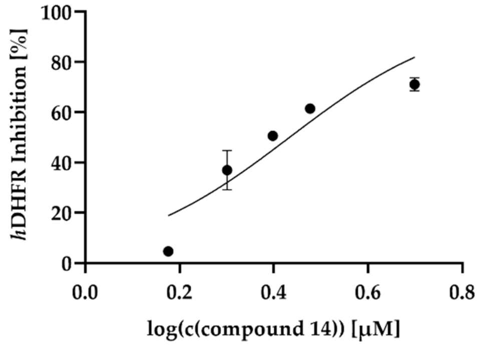

(**e**)

Figure S34. Determination of the IC_{50}/EC_{50} values of compound **14** against *Tb*PTR1 (**a**), *Tb*DHFR (**b**), *Lm*PTR1 (**c**), *Lm*DHFR (**d**) and *h*DHFR (**e**) through nonlinear regression analysis using the software GraphPad Prism 8 (Table 1).

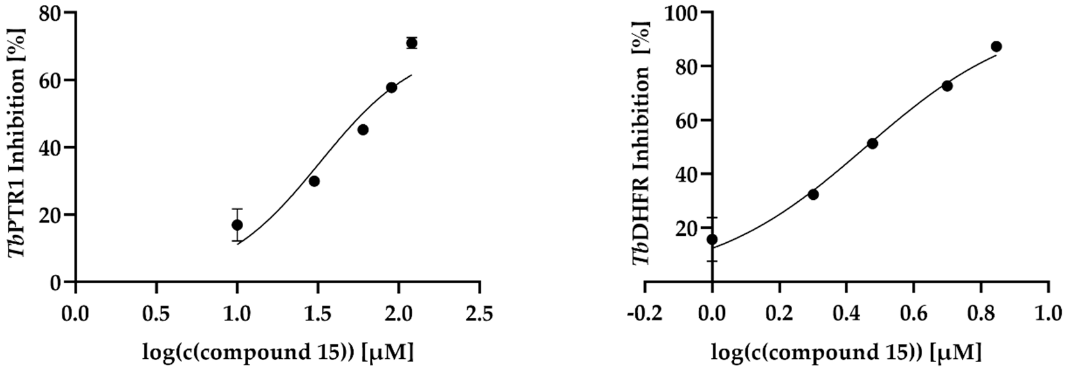

(**a**) (**b**)

Figure S35. Determination of the IC_{50}/EC_{50} value of compound **15** against *Tb*PTR1 (**a**) and *Tb*DHFR (**b**) through nonlinear regression analysis using the software GraphPad Prism 8 (Table 1).

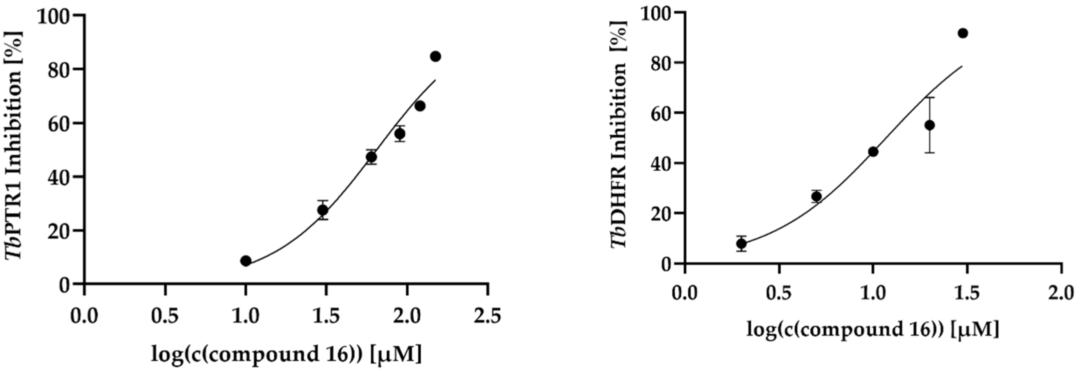

(**a**) (**b**)

Figure S36. Determination of the IC_{50}/EC_{50} value of compound **16** against *Tb*PTR1 (**a**) and *Tb*DHFR (**b**) through nonlinear regression analysis using the software GraphPad Prism 8 (Table 1).

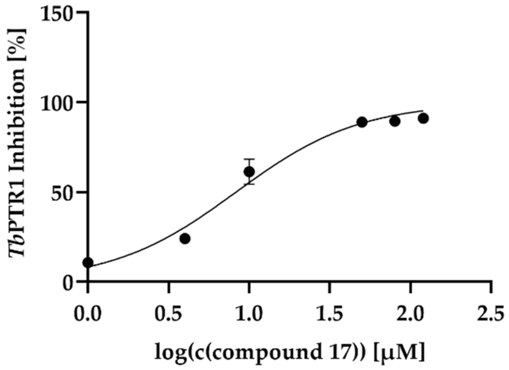

(**a**)

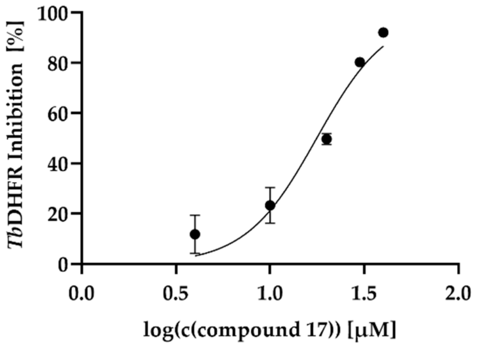

(**b**)

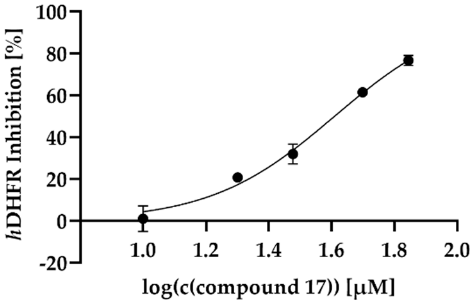

(**c**)

Figure S37. Determination of the IC_{50}/EC_{50} values of compound **17** against *Tb*PTR1 (**a**), *Tb*DHFR (**b**) and *h*DHFR (**c**) through nonlinear regression analysis using the software GraphPad Prism 8 (Table 1).

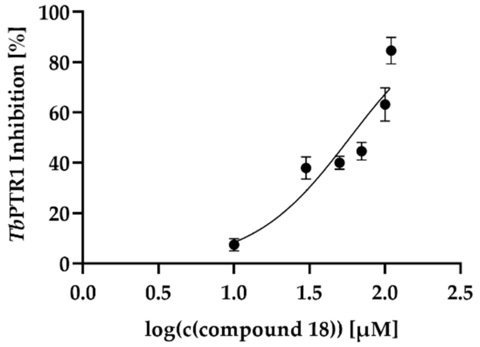

Figure S38. Determination of the IC_{50}/EC_{50} value of compound **18** against *Tb*PTR1 through nonlinear regression analysis using the software GraphPad Prism 8 (Table 1).

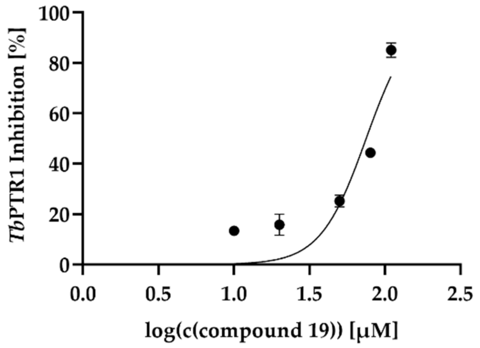

Figure S39. Determination of the IC_{50}/EC_{50} value of compound **19** against *Tb*PTR1 through nonlinear regression analysis using the software GraphPad Prism 8 (Table 1).

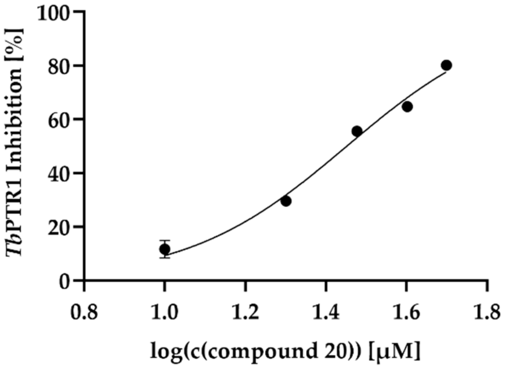

(**a**)

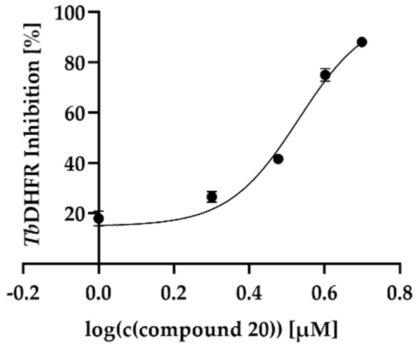

(**b**)

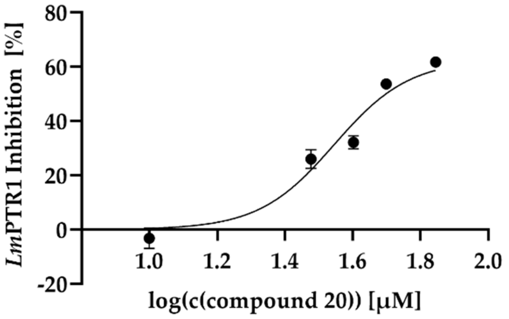

(**c**)

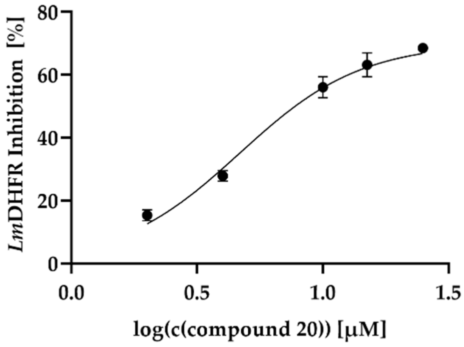

(**d**)

Figure S40. Determination of the IC_{50}/EC_{50} values of compound **20** against *Tb*PTR1 (**a**), *Tb*DHFR (**b**), *Lm*PTR1 (**c**) and *Lm*DHFR (**d**) through nonlinear regression analysis using the software GraphPad Prism 8 (Table 1).

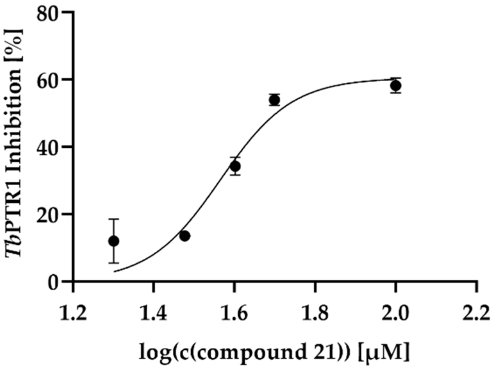

(**a**)

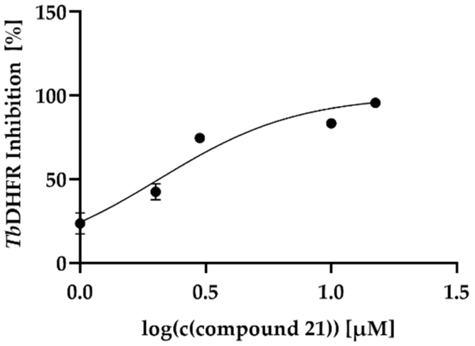

(**b**)

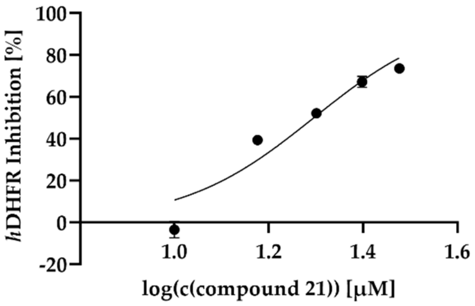

(**c**)

Figure S41. Determination of the IC_{50}/EC_{50} values of compound **21** against *Tb*PTR1 (**a**), *Tb*DHFR (**b**) and *h*DHFR (**c**) through nonlinear regression analysis using the software GraphPad Prism 8 (Table 1).

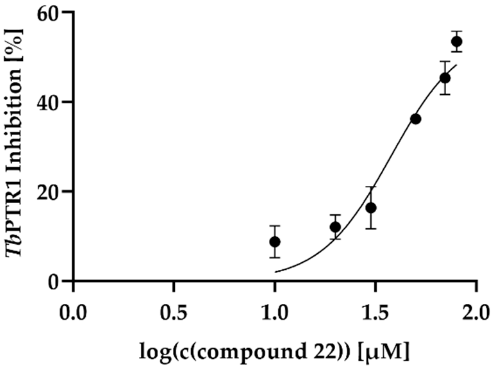

(**a**)

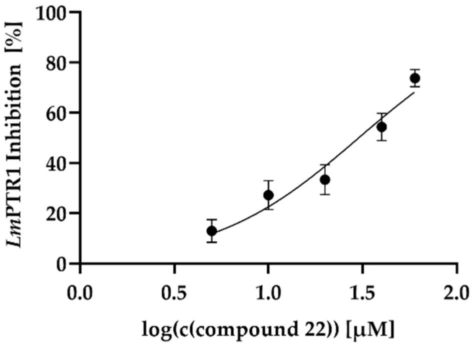

(**b**)

Figure S42. Determination of the IC_{50}/EC_{50} values of compound **22** against *Tb*PTR1 (**a**) and *Lm*PTR1 (**b**) through nonlinear regression analysis using the software GraphPad Prism 8 (Table 1).
